# Non-alcoholic fatty liver disease: relation to juvenile obesity, lipid profile, and hepatic enzymes

**DOI:** 10.25122/jml-2022-0091

**Published:** 2023-01

**Authors:** Raghid Reyadh Altalebi, Hany Akeel Al-hussaniy, Zahraa Salam Al-tameemi, Mohammed Abdul-Hassan AL-Zobaidy, Ali Hikmat Albu-Rghaif, Hayder Mutter Alkuraishy, Gomaa Mostafa Hedeab, Faizul Azam, Ali Mahmoud Al-Samydai, Meena Akeel Naji

**Affiliations:** 1 Department of Family Physician (CCFP), Madigan Medical Centre, Calgary, Alberta, Canada; 2 Department of Pharmacy, Bilad Alrafidain University College, Diyala, Iraq; 3 Dr. Hany Akeel Institute, Iraqi Medical Research Center, Baghdad, Iraq; 4 Department of Pharmacology, College of Medicine, Baghdad University, Baghdad, Iraq; 5 Department of Pharmacy, Ashur University College, Baghdad, Iraq; 6 Department of Clinical Pharmacology, College of Medicine, Almustansria University, Baghdad, Iraq; 7 Pharmacology Department & Health Research Unit, Medical College, Jouf University, Jouf, Saudi Arabia; 8 Pharmacology Department, Faculty of Medicine, Beni-Suef University, Beni-Suef, Egypt; 9 Department of Pharmaceutical Chemistry and Pharmacognosy, Unaizah College of Pharmacy, Qassim University, Uniazah, Saudi Arabia; 10 Pharmacological and Diagnostic Research Centre, Faculty of Pharmacy, Al-Ahliyya Amman University, Amman, Jordan

**Keywords:** non-alcoholic fatty liver disease, alanine transaminase, body mass index, liver cirrhosis, ALT – Alanine aminotransferase, AST – Aspartate aminotransferase, BMI – body mass index, CHOL – cholesterol, FBG – fasting blood glucose, GGT gamma-glutamyl transferase, LDL – Low-density lipoprotein, NAFLD – Non-alcoholic fatty liver disease, NASH – non-alcoholic steatohepatitis, TGL – Triglycerides

## Abstract

The prevalence of juvenile obesity is increasing, reaching epidemic proportions, presenting a link not only to NAFLD (non-alcoholic fatty liver disease) but to abnormal lipid profiles and liver enzyme abnormalities. Liver ultrasonography is a sensitive and specific tool for the recognition of NAFLD. This study aims to assess the association between NAFLD and juvenile obesity and to determine the other related changes in a set of indicators, including lipid profile abnormalities and serum transaminases. The sample included 470 obese and 210 non-obese individuals aged 6–16. Anthropometric measures were assessed, with the serum lipid profile and liver transaminases, and abdominal ultrasonography was used to detect NAFLD. Fatty liver was found in 38% of the obese subjects and none of the non-obese subjects. Within obese subjects, mean body mass index (BMI) and waist circumference increased significantly in patients with NAFLD compared to those without fatty liver. Moreover, LDL (low-density lipoprotein), CHOL (cholesterol), and serum liver enzymes were significantly higher in the presence of NAFLD. In conclusion, NAFLD commonly associates with juvenile obesity, relating to obesity and the abnormal lipid profile (including elevated CHOL and LDL) among obese people, reflecting elevated liver transaminases, which increase the risk of cirrhosis.

## INTRODUCTION

According to the reference range, juvenile obesity is the increased body weight in young people with a body mass index (BMI) above the 95^th^ percentile. This disease is rapidly increasing in prevalence, reaching epidemic proportions, and becoming one of the most challenging dilemmas pediatricians face [1]. Juvenile obesity is not only linked to obesity in adulthood and its related morbidity but is also associated with health risks for young people, including dyslipidemia, hyperinsulinemia, hypertension, and psychosocial problems [1, 2].

Non-alcoholic fatty liver disease (NAFLD) refers to a group of diseases that vary from basic hepatic steatosis to non-alcoholic steatohepatitis (NASH) [3]. Additionally, NAFLD varies from microvascular steatosis (simple steatosis) to macrovesicular or mixed macro-and-microvesicular steatosis with inflammatory signs and damage in the cellular compartment [4]. Although isolated fatty liver is benign, NASH is a severe liver disease that can develop into liver fibrosis or cirrhosis [5, 6].

NAFLD is usually related to obesity and is one of the most commonly emerging liver diseases in many countries, coinciding with the increased prevalence of obesity worldwide. The gold standard for diagnosing NAFLD is a liver biopsy; however, this procedure is not routinely performed in children due to its invasiveness and increased costs. Liver ultrasonography, although not sensitive enough to assess liver fibrosis or inflammation, has a sensitivity and specificity of around or over 90% [7, 8]. The recognition of NAFLD is of utmost importance, as the development of the disease depends on fat buildup in the liver. As a result, the value of non-invasive imaging in diagnosing fatty liver is apparent [6]. Increased transaminase levels in the blood have been linked to NAFLD both before and after puberty [7]. Despite this, the United States has a higher rate of liver damage with alanine aminotransferase (ALT) than with biochemical markers [8]. Therefore, it is vital to diagnose and start the treatment of NAFLD as early as possible to prevent irreversible liver damage. The first line of therapy consists mainly of lifestyle modifications, and the landmark of treatment is losing weight and improving insulin resistance, often through a multimodal approach [9].

Obese children may suffer abnormal lipid metabolism and/or abnormal serum lipid levels. Increased synthesis of very-low-density lipoproteins (VLDL) and triglycerides (TGL) are common features of obese children, along with high serum cholesterol (CHOL). Blood lipid profile changes, including increased TGL, can be associated with NAFLD; therefore, juvenile obesity might be associated with transaminases, lipid profile alterations, and an increased risk of steatosis [10]. Nonetheless, there is still controversy over the relationship between NAFLD and lipid profile among obese children.

Consequently, the objectives of our research are (1) to evaluate the correlation between NAFLD and juvenile obesity, (2) to highlight abnormalities in the lipid profile among obese children, and (3) to determine the relationship between obesity, abnormalities in the lipid profile, serum transaminases and NAFLD [11].

## MATERIAL AND METHODS

### Subjects

The sample included 470 obese (non-syndromic) and 210 non-obese individuals aged 6–16 years recruited from the WellCare Medical Clinic (outpatient clinic) in Alberta, Canada, from March 2016 to July 2020. All subjects were not presenting other affections. Obesity was defined as having a body mass index (BMI) greater than 95 percentiles based on gender and age.

### Anthropometric measurements

An electronic weight scale was used to evaluate body mass to the nearby 100 mg (patient barefoot and wearing light indoor clothes) (Model 770; Seca, Hamburg, Germany). A wall-mounted stadiometer with a ruler measured the standing height to the closest 0.5 cm. The waist circumference (WC) was evaluated around the thinnest section of the trunk, and the hip circumference (HC) around the gluteus greatest protrusion. We estimated the waist-to-hip ratio (WHR), defined as the waist perimeter divided by the hip perimeter. BMI was evaluated by subdividing their body mass (kg) by the square of their tallness (in meters). By international standards, a person is considered obese if the BMI is more than the average BMI of the population (50^th^ percentile for age and sex) [12, 13].

### Biochemical measurements

The blood samples were taken in the morning after more than 8 hours of fasting. Then serum lipid profile (CHOL, LDL, and TGL in mmol/L) and the fasting blood glucose (FBG) were estimated.

### Fatty liver

The presence of steatosis was determined by the high echogenicity of the liver tissue with densely packed tiny echoes. A radiologist was responsible for the interpretation of data and reporting.

### Exclusion criteria

Other primary liver disorders that might cause steatoses, such as alpha 1-antitrypsin deficiency, infectious hepatitis, or Wilson disease, were detected by clinical signs, symptoms, and specific disease markers. Chronic or recent drug or alcohol intake was considered as an exclusion criterion.

### Statistical analysis

All analyses were conducted using SPSS Inc, version 23, Chicago, IL. The differences between obese and non-obese participants in the measured variables were assessed using independent t-tests, similar to the assessment of obese subjects with and without fatty liver. The Chi-square test was also conducted. The relationships between total body composition, fat occurrence, and serum variables were assessed using bivariate correlation coefficients and age-adjusted partial correlations. The statistical significance level was reported at p<0.05.

## RESULTS

Descriptive data for body composition, central obesity, and serum variables for non-obese and obese subjects are shown in Table 1. There was no significant difference in the mean age (p=0.45) and gender distribution (p=0.49) of obese subjects compared to non-obese subjects. Obese participants had significantly higher weight (the percentage above ideal BMI), waist circumference, hip circumference (p=0.000), and WHR (P=0.0001) than non-obese participants. TGL, LDL, CHOL, ALT, and AST were significantly elevated in obese subjects (p-value between ≤0.01 and 0.000) compared to the controls. At the same time, height was not significantly different between the two groups. Twenty-six percent of obese subjects had high levels of LDL. In 49% and 17% of obese subjects, hypercholesteremia and hypertriglyceridemia were found, respectively. Approximately one-third of obese subjects had elevated ALT and AST, and high FBS was found in 11% of obese subjects. However, none of the non-obese subjects had elevated serum lipids, liver enzymes, or fasting blood sugar.

**Table 1:** Demographic and clinical characteristics of non-obese and obese subjects.

Variable	Non-obese N=210 mean±SD (Range)	Obese N=470 mean±SD (Range)	P-value*
**Age (year)**	10.76±1.66 (6–15)	11.36±1.94 (6–16)	0.45
**Sex** Male Female	120 (57.1) 90 (42.9)	310 (66) 160 (34)	0.49
**Height (cm)**	145.10±14.73 (114–175)	149.96±13.47 (116–181)	0.28
**Weight (kg)**	40.90±5.39 (21–61)	69.49±15.95 (26–112)	0.000 (HS)
**BMI (kg/m^2^)**	17.24±2.28 (12–23)	27.95±3.69 (18–45)	0.000 (HS)
**Waist circumference (cm)**	68.14±4.88 (51–82)	90.40±15.68 (56–126)	0.000 (HS)
**Hip circumference (cm)**	82.90±12.99 (55–113)	103.38±15.27 (68–139)	0.000 (HS)
**WHR**	0.82±0.04 (0.64–0.99)	0.87±0.07 (0.68–1.11)	P<0.0001
**TGL (mmol/l)**	0.79±0.13 (0.51–1.19)	1.67±0.25 (1.01–3.12)	P<0.0001
**HDL (mmol/l)**	1.1±0.12 (0.7–1.5)	0.9±0.14 (0.6–1.31)	P<0.0001
**LDL (mmol/l)**	2.55±0.41 (1.34–3.46)	3.53±1.02 (1.32–6.3)	P<0.0001
**CHOL (mmol/l)**	4.25±0.4 (3.36–5.3)	5.04±0.73 (3–7.4)	P<0.0001
**ALT (U/l)**	23.95±4.26 (11–40)	41.11±12.8 (11–89)	P<0.0001
**AST (U/l)**	22.67±4.44 (12–37)	38.91±11.2 (14–85)	0.001 (Sig)
**FBG (mmol/l)**	4.8±0.5 (3.4–5.9)	5±0.6 (3.6–6.8)	P<0.0001

* – Chi-square for sex and otherwise student t-test; HS – high significant P-value, Sig – significant P-value, WHR – waist-to-hip ratio.

Male and female obese participants were compared. No significant differences were found for any body composition, regional fat adiposity, or serum variables, so an analysis of both sexes was performed. BMI, hip circumference, waist circumference, and WHR were highly intercorrelated (age-adjusted r between 0.36 and 0.76). The correlation coefficients between BMI and hip circumference, waist circumference, and WHR were 0.57, 0.63, and 0.36, respectively. The correlations of the hip circumference with waist circumference and WHR were 0.76. The age-adjusted associations between total, regional adiposity, and serum variables are shown in Table 2. LDL and CHOL were moderately correlated with BMI (significant partial R=0.50) and weakly correlated with hip circumference (significant partial R=0.32 and 0.33, respectively). CHOL was weakly correlated with waist circumference (significant partial r=0.30), while TGL was poorly correlated with measures of adiposity. ALT and AST were only correlated with BMI with a significant partial R=0.37.

**Table 2:** Partial correlation coefficients for total body composition and regional adiposity with serum variables adjusted for age (obese children).

	TGL	LDL	CHOL	ALT	AST	FBG
**Hip**	-0.06	0.32	0.33	0.14	0.22	0.27
**Waist**	0.09	0.24	0.30	0.18	0.18	O.38
**WHR**	0.21	0.01	0.09	0.11	0.03	0.27
**BMI**	0.15	0.50	0.50	0.37	0.37	0.34

WHR – waist-to-hip ratio; BMI – body mass index.

NAFLD was found in 180 of 470 obese participants (38%) and none non-obese participants. Within obese subjects, the mean weight, BMI, percent above ideal BMI, and waist circumference were significantly higher in subjects with NAFLD than in those without (Table 3). LDL and CHOL were significantly higher in the presence of fatty liver, as were serum liver enzymes and fasting blood sugar. Among the group of NAFLD (N=180), 150 patients had a high CHOL (83.3%), 100 had high LDL (55.6%), and 40 had a high TGL (22.2%). This group also showed abnormally high levels of ALT and AST in 140 and 110 patients representing 77.8% and 61.1%, respectively. While the remaining obese subjects without NAFLD (n=290) had a high CHOL, LDL, and TGL in 25%, 6.3%, and 12.5%, respectively, and high ALT and AST in 12.5% and 6.3% of this group, respectively.

**Table 3:** Differences between subjects with non-fatty liver and subjects with NAFLD regarding demographic, whole body composition, and serum variables (obese children).

Variable	Non-fatty liver N=290 mean±SD (Range)	NAFLD N=180 mean±SD (Range)	P-value*
**Age (year)**	11.21±2.05 (6–16)	11.61±1.41 (6-15)	0.65
**Sex** Male Female	180 (58.1) 110 (68.8)	130 (41.9) 50 (31.3)	0.48
**Height (cm)**	147.41±10.8 (116–179)	154.06±11.41 (125–181)	0.18
**Weight (kg)**	63.07±15.7 (26–102)	79.83±13.85 (35–112)	0.009*
**BMI (kg/m^2^)**	28.34±2.14 (18–34)	32.56±5.1 (22–45)	0.002*
**Waist circumference (cm)**	86.66±11.1 (56–108)	96.44±12.59 (58–126)	0.04*
**Hip circumference (cm)**	100.28±10.42 (68–126)	108.39±9.67 (71–139)	0.08
**WHR**	0.86±0.06 (0.68–1.11)	0.89±0.05 (0.8–1.1)	0.30
**TGL (mmol/l)**	1.7±0.25 (1.01–2.46)	1.97±0.31 (1.01–3.12)	0.39
**HDL (mmol/l)**	0.99±0.12 (0.65–1.29)	0.96±0.14 (0.6–1.3)	0.47
**LDL (mmol/l)**	2.79±0.64 (1.32–4.5)	4.2±0.97 (1.8–6.3)	0**
**Chol (mmol/l)**	4.42±0.78 (3–5.8)	5.3±0.99 (3.15–7.4)	0.000**
**ALT (U/l)**	29.14±4.04 (11–44)	60.39±9.20 (22–89)	0.000**
**AST (U/l)**	30.03±6.11 (14–66)	53.22±8.44 (22–85)	0.000**
**FBG (mmol/l)**	4.8±0.6 (3.8–5.7)	5.4±0.8 (3.8–16.8)	0.006*

WHR – waist-to-hip ratio; BMI – body mass index.

Sensitivity and specificity with a 95% confidence interval of liver enzymes in the diagnosis of fatty liver were shown in Table 4. ALT and AST tests have higher specificity in diagnosing fatty liver and less sensitivity.

**Table 4:** Sensitivity and specificity and 95% confidence interval (CI) of liver enzymes for diagnosis of NAFLD.

	NAFLD No.	Non-fatty liver No.	Total	Sensitivity (95% CI)	Specificity (95% CI)
**ALT** High Normal Total	140 40 180	20 480 500	160 520 680	77.8 (66.0-86.8)	96 (87.4-99.1)
**AST** High Normal Total	110 70 180	40 460 500	150 530 680	61.1 (48.4-72.4)	92 (82.2-96.9)

## DISCUSSION

The prevalence of obesity and weight over the recommended standard has dramatically increased among children over the past 50 years [5, 12]. NAFLD is becoming more well-recognized among young people. Cirrhosis can develop in up to 15% of people, although the illness can be stopped by losing weight, which reduces fatty infiltration and reverses biochemical alterations [13–15].

In our study, the prevalence of NAFLD among the obese group was 38%. Various investigators have reported a prevalence ranging between 22.5% to 77% [6, 15]; this wide variability could be attributed to the degree and duration of obesity in the study populations as both research groups have utilized almost similar methodology depending on ultrasound for the diagnosis of NAFLD. NAFLD was not reported in any of the control group subjects. While most investigators consider obesity a major cause of NAFLD in the pediatric age group, a Japanese study involving 810 normal-weight children reported a prevalence of ultrasound fatty liver change in 2.6% of participants [16]. This finding could be related to a large study population. The present study has also shown an association between the severity of obesity as shown by body weight, BMI, and the prevalence of NAFLD, a finding agreed upon by some authors [17–19]. However, the disparity might be due to differences in illness diagnostic methods and patient population characteristics. The distribution of adipose tissue as evaluated by WHR to NAFLD could not be correlated; this is consistent with the finding of other researchers mentioning the poor reliability of WHR as an index for steatosis in childhood and puberty [20–22]. The degree of steatosis would be influenced by the distribution of adipose tissue, as determined by WHR, only near the conclusion of pubertal development. However, other investigators reported different results [23–25].

The present work showed a significant association between NAFLD and elevated CHOL, LDL, and CHOL/HDL but not with TGL. Other studies have also mentioned the elevated levels of free fatty acids among obese children with steatosis showing the importance of their assessment as part of the workup of obesity, especially with liver steatosis [26–28]. Some authors have related NAFLD to increased CHOL and LDL and elevated TGL [29]. This difference could be related to the distinction in food patterns between the study groups or the age variance, as TGL's relation to steatosis is mainly evident in late puberty only [30].

Our study showed significantly elevated ALT and AST levels among obese subjects compared to controls. Moreover, a highly significant association between NAFLD and elevated ALT and AST was found among the obese group. Other investigators have also shown an increase in hepatic transaminases among obese subjects [30]. Serum ALT is well known to be a marker of NAFLD in obese individuals without other identifiable causes for liver disease to the extent that it can be added to the US as a criterion for the diagnosis of NASH [31, 32]. Serum levels of transaminases (among which ALT is the most altered) positively correlate with the degree of steatosis and slight steatosis, which may not involve enzymatic modifications [33]. In our research, an elevated ALT was present in 77.8% of the obese with NAFLD, while AST was elevated only in 61.1% of patients. Other studies reported elevated enzymes in about 31.6% of their cases [11, 34]. The disparity might be explained by serum aminotransferases' inability to detect modest amounts of hepatic fat buildup; GGT appears to be useless in evaluating fatty liver in juvenile obesity, unlike the situation in adults where it is considered an important diagnostic marker [35, 36].

The cross-sectional design of our study limited our ability to examine the development of NAFLD and its association with anthropometric and biochemical markers over time. We cannot confirm if assumed NASH patients had aberrant histology since we do not have any liver biopsy data.

## CONCLUSION

NAFLD commonly associates with juvenile obesity. It is related to the degree of obesity and the abnormal lipid profile among obese people (including elevated CHOL and LDL) and can be reflected in elevated liver transaminases carrying out the risk of cirrhosis. Therefore, we recommend early detection of juvenile obesity through screening programs that should include US abdominal studies of obese children and, accordingly, early management of cases of NAFLD through dietary modifications, exercise, and drug therapy that can help prevent the risk of cirrhosis related to NAFLD later in life. Further, larger longitudinal studies, including children with various degrees of obesity, are also required to learn more about the natural course of NAFLD in obese children.
